# Using PaCO2 values to grade obesity-hypoventilation syndrome severity: a retrospective study

**DOI:** 10.1186/s40248-017-0093-4

**Published:** 2017-05-18

**Authors:** Mario Francesco Damiani, Vito Antonio Falcone, Pierluigi Carratù, Cristina Scoditti, Elioda Bega, Silvano Dragonieri, Alfredo Scoditti, Onofrio Resta

**Affiliations:** 1 Department of Respiratory Diseases, San Camillo Clinic, Taranto, Italy; 2 Department of Respiratory Diseases, San Paolo Hospital, Bari, Italy; 3 Institute of Respiratory Disease, University of Bari, Bari, Italy

**Keywords:** OHS, Obesity, Grading OHS

## Abstract

**Background:**

To date, an important aspect that has still not been clarified is the assessment of OHS severity. The purpose of this retrospective study was to evaluate whether grading OHS severity according to PaCO_2_ values may be useful in order to provide a more definite characterization and targeted management of patients. In this regard, baseline anthropometric and sleep polygraphic characteristics, treatment options, and follow up outcomes, were compared between OHS patients with different degree of severity (as assessed according to PaCO_2_ values).

**Methods:**

Patients were classified into three groups, according to PaCO_2_ values: 1) mild (46 mmHg ≤ PaCO_2_ ≤ 50 mmHg), moderate (51 mmHg ≤ PaCO_2_ ≤ 55 mmHg), severe (PaCO_2_ ≥ 56 mmHg). Therefore, differences among the groups in terms of baseline anthropometric, and sleep polygraphic characteristics, treatment modalities and follow up outcomes were retrospectively evaluated.

**Results:**

Patients with more severe degree of hypercapnia were assessed to have increased BMI and bicarbonate levels, worse diurnal and nocturnal hypoxemia, and a more severe impairment in pulmonary mechanics compared to milder OHS. CPAP responders rate significantly decreased from mild to severe OHS. After follow up, daytime sleepiness (as measure by the ESS), PaO_2_, and PaCO_2_ significantly improved with PAP therapy in all three groups.

**Discussion and Conclusions:**

Classification of OHS severity according to PaCO_2_ levels may be useful to provide a more defined characterization and, consequently, a more targeted management of OHS patients. Further studies are needed to confirm our findings.

## Background

Obesity Hypoventilation Syndrome (OHS) is defined by the association of obesity (body mass index [BMI] ≥ 30 kg/m^2^), and daytime hypercapnia (PaCO_2_ > 45 mmHg), in the absence of all other causes of alveolar hypoventilation (lung, neuromuscular, and chest wall diseases) [1]. Approximately ninety percent of patients with OHS exhibit obstructive sleep apnea (OSA), whereas ten percent of OHS subjects present with pure sleep hypoventilation [2–4]. To date, an important aspect that has not been clarified yet is the assessment of OHS severity; indeed, although some approaches to quantify the severity of OHS have recently been proposed [5], defined criteria to grade OHS severity are not reported in the literature. Therefore, in the present study, OHS patients were classified according to the degree of hypercapnia. The purpose of this retrospective study was to evaluate whether grading OHS severity according to PaCO_2_ values may be useful in order to provide a more definite characterization and targeted management of patients. In this regard, baseline anthropometric and sleep polygraphic characteristics, treatment options, and follow up outcomes, were compared between OHS patients with different degree of severity (as assessed according to PaCO_2_ values).

## Methods

### Patients

From March 2006 to April 2015, OHS was diagnosed in 109 clinically stable patients. These patients met the following criteria: obesity (BMI ≥ 30 kg/m^2^); daytime hypercapnia (PaCO_2_ > 45 mmHg); absence of any respiratory disorder that could be responsible for alveolar hypoventilation (lung, neuromuscular, and chest wall diseases); no evidence of acute respiratory failure (ie, patients with worsening symptoms during the last 2 weeks, a breathing frequency of > 30 breaths/min, a pH of < 7.35); and a follow up period of ≥ 6 months. Eleven subjects rejected positive airway pressure (PAP) treatment, and unfortunately were not followed up; fourteen subjects accepted PAP treatment, but never returned for follow up visit. The remaining 84 subjects accepted PAP therapy and were followed up for at least 6 months. These patients were classified into three groups, according to PaCO_2_ values: 1) mild (46 mmHg ≤ PaCO_2_ ≤ 50 mmHg), moderate (51 mmHg ≤ PaCO_2_ ≤ 55 mmHg), severe (PaCO_2_ ≥ 56 mmHg). Cabrera-Lacalzada and Diaz-Loboto have proposed grading patients with OHS as mild, moderate or severe based on five factors: PaCO_2_, PaO_2_, BMI, AHI, and complications/comorbidities [5]. According to their suggestion, we classified OHS into three groups of severity. On the other hand, we considered that the above mentioned five variables, taken together, could lead confusion; thus, we graded OHS according to only one parameter: PaCO_2_, due to its key diagnostic role. Furthermore, cutoff values among groups were closer than those suggested by Cabrera-Lacalzada and Diaz-Loboto, in order to provide a more definite characterization of patients. Therefore, differences among the groups in terms of baseline anthropometric and sleep polygraphic characteristics, treatment modalities, and follow up outcomes were retrospectively evaluated.

Institutional review board approval and patient consent were not necessary due to the retrospective nature of this study.

### Measurements

Daytime sleepiness was evaluated according to an Epworth Sleepiness Scale (ESS) questionnaire. Arterial blood gas levels were measured while patients were awake, sitting, and breathing room air. Spirometry and plethysmography were measured according to the European Respiratory Society recommendations [6].

Patients underwent diagnostic in-laboratory portable monitoring using: HypnoPTT, Somnea or Somtè. An apnea-hypopnea index (AHI) ≥5 was necessary to diagnose OSA. Sleep time was obtained as follows: each patient filled out a sleep diary, and hours in which patients reported they had not slept were subtracted from total hours of recording time. Outpatient sleep parameters were derived from the presumed sleep time (or useful recording time) [7].

### Positive airway pressure titration night during polysomnography (PSG)

PAP titration night was performed using a Compumedics E-Series polisomnography system. All subjects initially underwent continuous positive airway pressure (CPAP) titration, and the CPAP adjustement was performed with manual titration. Patients who achieved O_2_ saturation ≥ 90%, together with resolution of obstructive events (CPAP responders), were prescribed CPAP therapy at the effective pressure level; on the other hand, patients who presented persistent O_2_ desaturation despite disappearance of apneic-hypopneic events or persistent obstructive events despite high CPAP values (CPAP nonresponders), were switched to bilevel ventilation. Oxygen was added if required.

### Follow up

All patients underwent a medically supervised dietary regimen. In selected patients, a weight-reduction surgical intervention was performed. During each follow up visit, the Epworth Sleepiness Scale, pulmonary function testing, and arterial blood gas measurements were performed. CPAP/bilevel usage data were downloaded from the device.

### Statistical analysis

Data are presented as mean ± standard deviation (SD) unless otherwise indicated. Differences between two groups were analyzed by a Student’s *t*-test for independent samples. Differences among three groups were analyzed by analysis of variance with Bonferroni correction. The chi-square test was used to compare proportions between groups. Correlation were described with the Pearson correlation coefficient (r). A value of *p* < 0.05 was considered statistically significant. The analyses were made using STATISTICA 6.1 software (StatSoft Inc., Tulsa, Oklahoma).

## Results

Out of the 109 OHS patients who were included in this study, eleven (5 mild, 5 moderate, and 1 severe) did not agree to undergo in-laboratory positive airway pressure titration night during PSG; unfortunately, these subjects were not followed up. In addition, fourteen subjects (7 mild, 5 moderate, 2 severe) initially accepted CPAP or bilevel ventilation treatment, but never returned for follow up visit. The remaining 84 subjects received CPAP/bilevel ventilation treatment, and were followed up for at least 6 months. Baseline demographic and sleep polygraphic characteristics of patients who were excluded from the analysis were similar to the study population (data not shown).

### Baseline anthropometric variables

Baseline characteristics of study population, including age, sex, BMI, ESS scores, blood gas, and pulmonary function values, and comorbidities are shown in Table 1. BMI was significantly higher in severe OHS than in mild ones. PaCO_2_, and HCO3- were significantly higher in moderate, and severe OHS than in mild ones; in addition, PaCO_2_, and HCO3- in severe OHS were significantly higher compared to moderate OHS. PaO_2_ was significantly lower in moderate, and severe OHS than in mild ones; furthermore, PaO_2_ in severe OHS was significantly lower compared to moderate OHS. Forced expiratory volume in 1 s (FEV_1_), and forced vital capacity (FVC) were significantly lower in severe OHS than in mild ones.

**Table 1:** Baseline anthropometric characteristics of study population

	Mild OHS^a^ (*n* = 46)	Moderate OHS^b^ (*n* = 24)	Severe OHS^c^ (*n* = 14)
Age, yrs	55 ± 9.3	52.2 ± 11.1	55.7 ± 6.8
Sex, male/female	33/13	19/5	10/4
BMI, kg/m^2^	41.5 ± 8.2	45.1 ± 7.3	48.7 ± 6.5^#^
ESS	11.7 ± 5	13.7 ± 5.8	14.5 ± 4.5
pH	7.40 ± 0.03	7.39 ± 0.04	7.39 ± 0.02
PaO_2_, mmHg	72.6 ± 6.8	66.2 ± 6.5*	58 ± 6.2**^§^
PaCO_2_, mmHg	46.8 ± 1	52.3 ± 1.1*	57.6 ± 1.8**^§^
HCO3-, mmol/L	29.4 ± 1.7	33.4 ± 2.4*	36.8 ± 2**^§^
FEV_1_, % predicted	83.7 ± 15.3	76.4 ± 16.3	71.4 ± 11.5^#^
FVC, % predicted	85.1 ± 13.9	78.8 ± 15.6	73.9 ± 10.9^#^
FEV_1_/FVC ratio, %	80.4 ± 6.7	80 ± 4.1	79.4 ± 4
TLC, % predicted	90 ± 11.6	87.2 ± 16.2	82.9 ± 12.1
Hypertension, *n* (%)	35 (76)	19 (79)	11 (78)
Diabetes mellitus, *n* (%)	16 (34)	11 (45)	8 (57)
Dyslipidemia, *n* (%)	13 (28)	8 (33)	6 (42)
Current smokers, *n* (%)	11 (23)	10 (41)	4 (28)
Ex smokers, *n* (%)	19 (41)	4 (16)	5 (35)

Data are presented as mean values ± SD unless otherwise indicated

*OHS* Obesity-Hypoventilation Syndrome, *BMI* body mass index, *ESS* Epworth Sleepiness Scale, *FEV*_*1*_ forced expiratory volume in 1 s, *FVC* forced vital capacity, *TLC* total lung capacity

^#^ *P* < 0.05 severe vs mild OHS; *P < 0.01 moderate vs mild OHS; **P < 0.01 severe vs moderate OHS; ^§^ *P* < 0.01 severe vs mild OHS

^a^46 mmHg ≤ PaCO_2_ ≤ 50 mmHg

^b^51 mmHg ≤ PaCO_2_ ≤ 55 mmHg

^c^PaCO_2_ ≥ 56 mmHg

### Sleep variables

All OHS patients were also diagnosed with OSA (AHI ≥ 5). Sleep variables of study population are shown in Table 2. Total sleep time with oxyhemoglobin saturation below 90% (TST90) was significantly higher in moderate and severe OHS than in mild ones; in addition, TST90 in severe OHS was significantly increased compared to moderate OHS. Mean arterial oxygen saturation (SaO_2_) was significantly lower in moderate, and severe OHS than in mild ones. SaO_2_ nadir was significantly lower in severe OHS than in mild ones. No difference was found among groups in terms of AHI. Diurnal PaCO_2_ was significantly correlated to TST90 (*r* = 0.22; *p* = 0.03), mean SaO_2_ (*r* = − 0.27; *p* = 0.01), and SaO_2_ nadir (*r* = −0.25; *p* = 0.02); no significant correlation was found between diurnal PaCO_2_ and AHI (*r* = 0.17; *p* = 0.08).

**Table 2:** Sleep variables of study population

	Mild OHS^a^ (*n* = 46)	Moderate OHS^b^ (*n* = 24)	Severe OHS^c^ (*n* = 14)
AHI, events/h	65.4 ± 17.8	73.8 ± 16.5	70.7 ± 17.6
TST90, %	37.8 ± 21.2	55.5 ± 19.4*	86.3 ± 24.5**^§^
Mean SaO_2_, %	89.3 ± 3.3	86.6 ± 3.9*	84.1 ± 2.8^§^
SaO_2_ nadir, %	68.5 ± 11.7	63.4 ± 12.5	59.2 ± 10.7^#^

Data are presented as mean values ± SD unless otherwise indicated

*OHS* Obesity-Hypoventilation Syndrome, *AHI* apnea-hypopnea index, *TST90* total sleep time with oxyhemoglobin saturation below 90%, *SaO*_*2*_ arterial oxygen saturation

^a^46 mmHg ≤ PaCO_2_ ≤ 50 mmHg

^b^51 mmHg ≤ PaCO_2_ ≤ 55 mmHg

^c^PaCO2 ≥ 56 mmHg

^#^ *P* < 0.05 severe vs mild OHS; *P < 0.01 moderate vs mild OHS; **P < 0.01 severe vs moderate OHS; ^§^ *P* < 0.01 severe vs mild OHS

### Treatment modalities

**Fig. 1:**
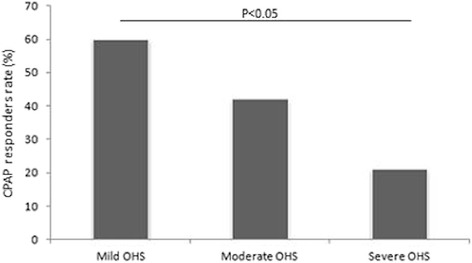
CPAP responder rates among mild, moderate, and severe OHS

CPAP responder rates among mild, moderate, and severe OHS are reported in Fig. 1. In mild OHS group, 60% of subjects (28/46) were CPAP responders; in moderate OHS group, CPAP responders rate was 42% (11/26), which was slightly but not significantly reduced compared to mild OHS; in severe OHS group, CPAP responders rate was 21% (3/14), which was slightly but not significantly reduced compared to moderate OHS, but was significantly decreased compared to mild OHS. Mean effective pressures were similar among mild, moderate, and severe OHS (12.8 ± 1.9 cmH_2_O, 13.3 ± 1.4 cmH_2_O, 12.6 ± 0.5 cmH_2_O, respectively; *p* = nonsignificant). CPAP nonresponders were set on bilevel positive pressure ventilation; with regard to bilevel users, mean inspiratory and expiratory therapeutic pressures were similar among mild, moderate, and severe OHS (mean inspiratory pressures: 19.2 ± 3.5 cmH2O, 20.6 ± 3 cmH2O, 20 ± 2.6 cmH_2_O, respectively; *p* = nonsignificant; mean expiratory pressures: 8.7 ± 2.4 cmH_2_O, 9.9 ± 2.1 cmH_2_O, 9.3 ± 2 cmH_2_O, respectively; *p* = nonsignificant). Eight subjects (3 mild, 2 moderate, 3 severe) received additional oxygen therapy. In most of cases, CPAP nonresponders were switched to bilevel therapy due to the presence of oxygen desaturation despite disappearance of obstructive events; however, in a few patients (2 mild, 1 moderate, 1 severe), the switch from CPAP to bilevel therapy was due to the persistence of obstructive events despite high CPAP values.

### Follow up

The mean follow up period was 55 ± 27 months. The mean ESS significantly improved in all three groups: in mild OHS, the ESS changed from 11.7 ± 5 at baseline to 5 ± 2.9 after follow up (*p* < 0.01); in moderate OHS, changed from 13.7 ± 5.8 to 5.3 ± 3.8 (*p* < 0.01); in severe OHS, changed from 14.5 ± 4.5 to 6.6 ± 3.5 (*p* < 0.01). In all three groups, the comparison of pretreatment with the last posttreatment measurements demonstrated significant improvements in both mean PaO_2_ values (mild: 72.6 ± 6.8 mmHg vs 75.5 ± 5 mmHg, *p* < 0.05; moderate: 66.2 ± 6.5 mmHg vs 71.1 ± 5.3 mmHg, *p* < 0.01; severe: 58 ± 6.2 mmHg vs 65.4 ± 4.2 mmHg, *p* < 0.01), and mean PaCO_2_ values (mild: 46.8 ± 1 mmHg vs 42.4 ± 2 mmHg, *p* < 0.01; moderate: 52.3 ± 1.1 mmHg vs 46.3 ± 2.2 mmHg, *p* < 0.01; severe: 57.6 ± 1.8 mmHg vs 49.1 ± 1.9 mmHg, *p* < 0.01). However, PaO_2_ and PaCO_2_ measured after follow up were significantly worse in moderate (PaO_2_ 71.1 ± 5.3 mmHg, PaCO_2_ 46.3 ± 2.2 mmHg), and severe OHS (PaO_2_ 65.4 ± 4.2 mmHg, PaCO_2_ 49.1 ± 1.9 mmHg) compared to mild ones (PaO_2_ 75.5 ± 5 mmHg, PaCO_2_ 42.4 ± 2 mmHg; *p* < 0.01 in all comparisons); in addition, PaO_2_ and PaCO_2_ measured after follow up were significantly worse in severe OHS compared to moderate ones (*p* < 0.01 in both comparisons).

No significant weight loss nor improvement in pulmonary function values were observed at the end of follow up period (data not shown). Two patients (1 mild, 1 moderate) underwent weight reduction surgery, and both of them lost a significant amount of weight and no longer required bilevel therapy. CPAP/bilevel usage was similar among groups (mild: 4.7 ± 1.1 h/night; moderate: 5.1 ± 1 h/night; severe: 5 ± 0.8 h/night; *p* = nonsignificant).

## Discussion

This is the first study, to our knowledge, in which OHS patients with different degree of severity (as assessed according to PaCO_2_ values) were compared in terms of baseline anthropometric and sleep variables, treatment modalities, and follow up outcomes. Recently, Cabrera Lacalzada et al. [5] proposed to assess OHS severity according to five variables, such as PaCO_2_, PaO_2_, BMI, AHI, and comorbidities. However, a classification based on many parameters may be difficult to implement and, in some cases, misleading. Therefore, we quantified OHS severity according to only one variable, such as PaCO_2_; indeed, in obese patients without coexisting lung/neuromuscular/chest wall diseases, PaCO_2_ is the only required parameter to distinguish between OHS and otherwise healthy obese subjects; thus, it is conceivable that hypercapnia levels may also be useful for distinguish among mild, moderate, and severe OHS.

With regard to baseline characteristics, patients with more severe degree of hypercapnia were found to have increased BMI and bicarbonate levels, worse diurnal and nocturnal hypoxemia, and a more severe impairment in pulmonary mechanics compared to milder OHS. Each of these results has its pathophysiological basis. Bicarbonate levels can increase due to metabolic compensation for chronic respiratory acidosis; indeed, bicarbonate is considered to be a clinical predictor of hypercapnia [8]. To date, it was demonstrated the association among hypercapnia, BMI levels, and degree of restrictive chest wall mechanics, among obese patients [9]. The effects of obesity on pulmonary mechanics are influenced by both the distribution and quantity of excess adipose tissue [10–12]. OHS subjects present central pattern of obesity [13]; central adiposity is responsible for cephalic displacement of the diaphragm, resulting in reduction of functional residual capacity, which is more marked in supine position; therefore, when a subject breathes at low lung volumes, small airway closure an air-trapping may occur, with consequent expiratory flow limitation and development of intrinsic positive end-expiratory pressure [14]. This inefficient mechanical performance is generally demonstrated on spirometry by low FVC and FEV_1_, and normal FEV_1_/FVC ratio [15]. It is likely that increased BMI and a more severe impairment in lung function may be partially responsible for higher hypercapnia levels. With regard to sleep variables, TST90 and mean SaO_2_ have been shown to be strongly associated with the onset of awake hypercapnia [9]. Similarly, in the present study, it was observed worse nocturnal (and diurnal) hypoxemia in more hypercapnic patients. Although to date it is not possible to know whether hypoxemia is the cause or consequence of hypercapnia, some authors recently showed that hypoxemia may interfere with the synthesis of neurotransmitters involved in central respiratory control [16, 17]. Interestingly, no significant differences among groups were observed in terms of AHI. On one hand, this result leads to consider this parameter as not suitable to distinguish among OHS subjects with different degree of severity. On the other hand, it should be remarked that all patients included in our study were diagnosed with OSA, and mean AHI value was really high (>50 events/h) in all three groups. This confirms the well known pathophysiological link between OSA and OHS [18–20].

In the present study, CPAP responders rate significantly decreased from mild to severe OHS. In this regard, it should be mentioned a landmark study, in which Rapoport et al. [21] introduced the concept of CPAP responder/nonresponder; CPAP responders were defined as those who achieved daytime eucapnia after effective treatment of OSA, whereas others, christened “true Pickwickians”, continued to hypoventilate despite resolution of obstructive events [21]. In the present study, CPAP nonresponders were generally switched to bilevel due to the persistence of O_2_ desaturation despite disappearance of apneas-hypopneas, which is a marker of sleep hypoventilation [22]. Our results indicate that OHS patients with a more severe degree of hypercapnia were more likely to be “true Pickwickians” compared to milder ones. Among CPAP responders, OSA is considered to be the primary mechanism responsible for the development of OHS [23, 24]; differently, pathogenesis in CPAP nonresponders may involve other mechanisms (such as abnormal pulmonary mechanics due to obesity, blunted respiratory drive, and leptin resistance), in addition to OSA [25]. Therefore, among “true Pickwickians”, OSA and the others mechanisms leading to hypercapnia may act sinergically, and this may explain the higher prevalence of CPAP nonresponders among severe OHS compared to milder ones. It is important to remark that CPAP nonresponders rate included also patients in whom obstructive events persisted despite high CPAP values. With regard to these patients, it is not possible to know whether they were “OSA-linked” OHS or “true Pickwickians”; however, the low and among-group similar number of this subset of patients may to some extent counterbalance this confounder.

After follow up, daytime sleepiness (as measure by the ESS), PaO_2_, and PaCO_2_ significantly improved with PAP therapy in all three groups. However, both PaO_2_ and PaCO_2_ measured after follow up were significantly worse in more severe OHS compared to milder ones; in addition, mean PaCO_2_ values were still > 45 mmHg in moderate-severe patients. Although these results confirm the effectiveness of PAP in OHS [4], they suggest the usefulness of a multidisciplinary approach to this disease, mostly in moderate-severe OHS.

It is important to highlight that this study was focused on clinically stable OHS, and therefore did not include subjects with acute type II respiratory failure. In this regard, recently Marik [26] conied the term “malignant” OHS to describe a subset of OHS patients with several life-threatening systemic complications of morbid obesity, and whose clinical presentation is characterized by acute on chronic hypercapnic respiratory failure. Although this subset of patients was not included in our study, it seems evident that they should be considered as severe OHS and treated with an aggressive multisystem approach.

This study has some limitations, which are due to its retrospective nature. First, multiple measurements of arterial blood gases were not available for more precise estimates of PaCO_2_ and PaO_2_ at baseline and follow up. Secondly, this study was limited by a wide range of follow up period. Thirdly, data regarding respiratory muscle performance, ventilator CO_2_ chemosensitivity, and nocturnal PaCO_2_ were not reported (indeed, these parameters are not routinely evaluated in our sleep clinic). Lastly, our study sample was not representative of all the OHS population, due to the absence of patients with pure sleep hypoventilation.

It is important to remark that, to our knowledge, it has been observed hypercapnia among OSA patients [15]. Therefore, the diagnosis of severe OSA in all studied subjects may have been a confounding factor for the purposes of the present study. On the other hand, it is important to highlight that all patients included in our work met the standardized diagnostic criteria for OHS, as reported in many previous studies [1, 3–5].

## Conclusions

OHS patients with more severe degree of disease (as assessed according to PaCO_2_) are characterized by increased BMI and bicarbonate levels, and a worsening in terms of diurnal PaO_2_, TST90, mean SaO_2_, SaO_2_ nadir, and pulmonary mechanics. In addition, severe OHS more often requires switch from CPAP to bilevel compared to mild ones. Furthermore, a multidisciplinary approach should be considered to manage OHS patients, mostly in moderate-severe ones. The results of the present study suggest that classification of OHS severity according to PaCO_2_ levels may be useful to provide a more defined characterization and, consequently, a more targeted management of OHS patients. Further studies are needed to confirm our findings.

## Abbreviations

AHI: Apnea-hypopnea index
BMI: Body mass index
CPAP: Continuous positive airway pressure
ESS: Epworth Sleepiness Scale
FEV_1_: Forced expiratory volume in 1 s
FVC: Forced vital capacity
OHS: Obesity-hypoventilation syndrome
OSA: Obstructive sleep apnea
PAP: Positive airway pressure
SaO_2_: Arterial oxygen saturation
SD: Standard deviation
TST90: Total sleep time with oxyhemoglobin saturation below 90%
